# Serosurveillance of infectious agents in equines of the Central Valley of Costa Rica

**Published:** 2014-11-16

**Authors:** D. Jiménez, J.J. Romero-Zuñiga, G. Dolz

**Affiliations:** Programa de Investigación en Medicina Poblacional, Escuela de Medicina Veterinaria, Universidad Nacional, P.O. Box 86-3000, Heredia, Costa Rica

**Keywords:** Costa Rica, Equines, Infectious agents, Risk factors, Seropositivity

## Abstract

Blood samples from 181 equines from the Central Valley of Costa Rica were collected in the year 2012 to determine the presence of antibodies against selected infectious agents in horses and to determine the risk factors associated with these agents. The presence of antibodies against Equine Infectious Anemia Virus (EIAV), Equine Herpes Virus 1 and 4 (EHV-1 and EHV-4), West Nile Virus (WNV), Influenza A Virus (IAV), Equine Viral Arteritis Virus (EVAV), *Babesia caballi*, *Theileria equi, Neospora caninum* and *Chlamydia abortus* was determined using commercial assays, and risk factors associated with seropositivity to the different infectious agents was established. The most seroprevalent agent detected was EHV-4 (96.7%), followed by WNV (44.2%), and IAV (41.8%). Horses >3 years, used for work or sports, and with access to pastures, had significantly increased probability to be seropositive to WNV, whereas horses used for breeding and recreational purposes, being stabled, and without access to pastures, had significantly greater probability to be seropositive to IAV. Seroprevalence to *B. caballi* (19.9%) was lower than to *T. equi* (38.1%). For *B. caballi*, access to pastures was determined as a risk factor, whereas being older than 3 years was established as a risk factor for *T. equi*. Low seroprevalences were determined for EHV-1 (5.0%), EVAV (5.0%), *C. abortus* (4.8%), and *N. caninum* (4.4%). Mares having history of abortion were more likely to be seropositive to EHV-1, whereas horses >3 years, used for work and sports, and mares having multiple parturitions, were more likely to be seropositive to *N. caninum*. None of the horses were seropositive to EIAV. Earlier, only diseases caused by EIAV, WNV and piroplasmosis were reported in Costa Rica. The present study however, determined the presence of carriers for EHV-1, EHV-4, and EIAV.

## Introduction

Infections due to Equine Infectious Anemia Virus (EIAV), Equine Herpes Virus 1 and 4 (EHV-1 and EHV-4), West Nile Virus (WNV), Influenza A Virus (IAV), Equine Viral Arteritis Virus (EVAV), *Babesia caballi*, *Theileria equi, Neospora caninum* and *Chlamydia abortus* are known to cause clinically important diseases in horses (Reed *et al.*, 2004).

EIAV is a retrovirus that causes a disease characterized by recurrent febrile episodes, anemia, rapid loss of weight and edema. All infected horses, including those that are asymptomatic, become carriers and are infectious for the rest of their life (Albayrak and Ozan, 2010). Equine herpesvirus infections in horses remain a significant cause of abortion, respiratory and neurologic disease. EHV-4 is considered to cause respiratory tract disease, known as rhinopneumonitis among horses worldwide, whereas EHV-1 seems responsible especially for abortions, neurologic and respiratory disease (Murphy *et al.*, 1999; Reed and Toribio, 2004).

The ability to establish latent infections with periodic reactivation and transmission to other horses is an important feature of these herpesviruses (Reed and Toribio, 2004). WNV is a member of the Japanese encephalitis serogroup within the family Flaviviridae and was introduced in 1999 to the American continent. This arbovirus is typically transmitted by *Culex* spp. mosquitoes that are opportunistic feeders and often transmit the viruses from their sylvatic or enzootic maintenance hosts to dead-end hosts, such as humans and horses.

Horses are exposed to numerous mosquito bites and may serve as sensitive sentinels for local arbovirus activity, which can be detected by screening for the novel production of specific virus-reactive antibodies. Horses infected with WNV may develop neurologic disease, sometimes leading to death, but most infections are subclinical and will only be detected through testing for seroconversion (Mattar *et al.*, 2011).

IAV is a major cause of respiratory disease in the horse. In non-vaccinated horses clinical signs typically include a harsh dry cough, labored breathing, loss of appetite, depression, and lethargy. Horses are also highly susceptible to secondary bacterial infections, which may result in severe pneumonia. In vaccinated horses, or those that have been previously infected, clinical signs are usually mild or non-apparent. Much effort in recent years has been put into studying antigenic variation, development of new vaccination strategies and, more recently, the factors that may be important for the development of disease. In the last decade, equine influenza virus crossed the species barrier establishing its presence in dogs in the United States of America (Elton and Bryant, 2011).

EVAV causes sporadic cases of respiratory disease and abortion, mares and geldings discharge the virus after acute infection, however 30% to 60% of stallions become persistently infected. In these animals, EVAV is maintained within the reproductive tract, and is shed continuously in the semen. Persistent infection with EVAV in stallions has no negative consequences for fertility, but mares inseminated with virus-contaminated semen can develop an acute infection. These mares then shed large amounts of virus in respiratory secretions and urine, leading to spread of the virus to other susceptible horses. Acute infection at later stages of gestation can lead to abortion (Glaser *et al.*, 1996).

Equine piroplasmosis is caused by *B. caballi* and *T. equi*, an endemic disease to most tropical and subtropical areas transmitted by ixodid ticks. The primary clinical outcome of acute infection is lysis of erythrocytes, and the clinical signs include fever, ventral edema, icteric sclera, pale mucous membranes, dark urine, anemia, weakness, lethargy, reduced feed intake and mild colic with reduced fecal output (Kappmeyer *et al.*, 2012; Laus *et al.*, 2013).

*N. caninum* is a protozoan parasite, which affects dogs as definitive hosts, and several mammalian species as intermediate hosts, mainly causing abortions and central nervous system disorders. Horses can serve as an intermediate host. Tachyzoites of *N. caninum* were first found in a fetus lung, whereas tachyzoites of *Neospora hughesi* were isolated from an adult horse (Dubey and Porterfield, 1990; Marsh *et al.*, 1998). In horses, neosporosis causes abortions, and neonatal, visceral and neurological disorders. *N. caninum* is related to abortions whereas *N. hughesi* to neurological disease (Leon *et al.*, 2012; Villalobos *et al.*, 2012). Recently, Dangoudoubiyam *et al*. (2011) detected anti-*Neospora* spp. antibodies in 3.5% horses from Costa Rica by using an NhSAG1 ELISA.

*C. abortus*, formerly *Chlamydophila psittaci* serotype 1, has been rarely detected in horses with acute respiratory infections until now (Theegarten *et al.*, 2008), where it was found in the lungs of both, clinically healthy horses and those with recurrent airway obstruction. Using molecular techniques, *C. abortus* was recently detected in a case of abortion (Leon *et al.*, 2012).

Epidemiological studies have been conducted in advanced countries, but there are only few reports of the prevalence of these agents in tropical countries (Sellon and Long, 2007) and in Costa Rica (Pineda, 1998; Dangoudoubiyam *et al.*, 2011; Hobson-Peters *et al.*, 2011; Vega, 2011; Posada, 2012; Fonseca-Salazar, 2013). The purpose of the present study was to determine the presence of antibodies to selected infectious agents in equines from provinces of the Central Valley from Costa Rica and to determine risk factors associated with these agents.

## Materials and Methods

### Size, type of sample and data collection

A cross-sectional survey of 181 equines living in the Central Valley of Costa Rica was carried out, to determine presence of antibodies against several infectious agents (EIAV, EHV-1, EHV-4, WNV, IAV, EVAV, *B. caballi, T. equi, N. caninum* and *C. abortus*). The formula described by Wayne (2002), using an expected prevalence of 50% (confidence level of 95% and error of 5%) was used. Selection of the animals for sampling was based on voluntary participation of the owners of the horses; inclusion criterion was that the animals had never been vaccinated against the infectious agents in study.

During 2012 a total of 181 blood samples were collected from horses belonging to the Central Valley of Costa Rica. The blood sample (2-4ml) was taken from the jugular vein of each equine and maintained at 4°C until arrival to the laboratory, serum was then separated and stored at -20°C until analysis. In addition, a questionnaire was used to collect general data, such as provenance (province), gender, age (less than 3 years and more than 3 years) and breed. Also, activity for which the horse was used (work, sports, breeding, and recreational purposes), and information about place of living and access to pastures was collected. Finally, the clinical history was inquired, giving special emphasis to respiratory (symptoms, treatment and evolution) and reproductive (abortions) diseases of the horses in the past.

### Serological analysis

All 181 sera were examined for the presence of antibodies to EIAV, EHV-1, EHV-4, WNV, EVAV, *B. caballi*, *T. equi* and *N. caninum*, and 146 were tested for antibodies to IAV and *C. abortus*, since the serum samples of some animals were not sufficient to perform all serological tests.

Serum samples were tested with commercial immunodiffusion (ID) or immunoenzymatic (ELISA) assays following the manufacturers´ instructions. The following assays were used: VMRD^®^ EIAV ID (sensitivity 99%, specificity 100%); SVANOVIR^®^ EHV-1/EHV-4 indirect ELISA; ID Screen^®^ West Nile competition ELISA; ID Screen^®^ Influenza A competition ELISA; ID Screen^®^ Equine Viral Arteritis indirect ELISA; VMRD^®^ *B. caballi* competition ELISA (sensitivity and specificity 100%); VMRD^®^ *B. equi* competition ELISA (sensitivity 95%, specificity 99.5%); VMRD^®^ *N. caninum* competition ELISA (sensitivity 96%, specificity 99%); and ID Screen^®^ *C. abortus* competition ELISA (sensitivity 100%, specificity 99.7%).

### Statistical analysis

Activity and for which the horses were used, were combined in two groups for statistical analysis. The first group comprised of horses used for recreational and breeding purposes, and the second group comprised of horses used for work and sports. Animals in the first group were moved more often to other areas (provinces) of the country, either through fairs or street exhibitions (“topes”), whereas animals in the second group remained mainly in the same area. Sporting events took place only in the province of San Jose.

Descriptive statistic about general characteristics of the horses and diseases comprised of frequencies, measures of central tendency (means, median) and dispersion (standard deviation).

To assess the relationships of independent variables with the diseases, the odds ratio (OR) rate ratio was calculated using unconditional logistic regression. The regression procedure consisted of two steps:

1) univariable analysis, and 2) multivariable analysis. In the second step, all variables with *P* <0.25 in the univariable analysis were included. A backward building model was followed based on the likelihood ratio test.

The process of exclusion-inclusion of each variable into the multivariable model tested confounding and interaction by comparison of the estimated coefficients in the new model with the estimated coefficients and likelihood ratio of the old model. Confounding was deemed present if at least one coefficient changed more than 10% (if the rate ratio had a value between 0.7 and 1.5) or if at least one coefficient changed more than 25% (if the rate ratio had a value < 0.7 or > 1.5).

Finally, variables that were excluded in the univariable step were checked on collinearity with the variables in the final model to check for potential confounding by calculation of simple correlations. The data was analyzed using EGRET 2.0 (Cytel Sotware Corp.)

## Results

The 181 equines samples were collected in the provinces of Alajuela (76), Cartago (5), Heredia (28) and San José (72). From the total, 98 (54.1%) were males (36 castrated), 83 (45.9%) were females, and 116 (64.1%) animals were older than 3 years old (66 males and 50 females). The main breed was represented by Costarricense de Paso (99 animals, 54.7%), followed by Iberoamericanos (31 animals, 17.1%) and Andalusian (11 animals, 6.1%), whereas 40 animals (22.1%) belonged to other breeds (Warmbloods, Arabians, and Quarter Horses).

Most of the animals (125, 69.1%) were used for recreational (119/125, 95.2%) and breeding purposes (6/125, 4.8%), and 56 (30.9%) for work (37/56, 66.1%) or sports (19/56, 33.9%). A total of 162 (89.5%) horses lived in stables (average of horses in stables: 50), the remaining animals lived on paddocks 19 (10.5%), and only 35 (19.3%) had access to paddocks, however all lived with other equines. The owners revealed respiratory disease (coughing and nasal secretions) in 14 (7.7%) animals, whereas 167 (92.3%) never noticed any respiratory symptom.

A total of 44.0% (22/50) of females in reproductive age had given birth, and only 8.0% (4/50) had suffered abortions. The results of the serological analysis of the horses to the different infectious agents are shown in Table 1.

**Table 1:** Presence of antibodies against selected infectious agents (EIAV, EHV-1, EHV-4, WNV, EVAV, IAV, *B. caballi, T. equi,* *N. caninum* and *C. abortus*) in equines from the Central Valley of Costa Rica.

Infectious agent	Seropositives/Total (%)
EIAV	0/181 (0)
EHV - 4	175/181 (96.7)
EHV - 1	9/181 (5.0)
WNV	80/181 (44.2)
IAV	61/146 (41.8)
EVAV	9/181 (5.0)
*B. caballi*	36/181 (19.9)
*T. equi*	69/181 (38.1)
*N. caninum*	8/181 (4.4)
*C. abortus*	7/146 (4.8)

Almost all animals (96.7%) were seropostive to EHV-4, the most prevalent infectious agent among horses, followed by WNV (44.2%), IAV (41.8%), and *T. equi* (38.1%). The risk factors determined for the different infectious agents are shown in Table 2.

**Table 2:** Risk factors determined for horses of the Central Valley from Costa Rica seropositive to different infectious agents.

Infectious agent	Variable	Risk Level	OR	95% CI	P
EHV-1	Abortion	Having history of abortion	15.00	0.31-797.81	0.027
WNV	Age	> 3 years	2.22	1.07-4.63	0.0196
	Activity	Work or sport	2.49	1.23-5.02	0.056
	Access to pastures	Access to pastures	2.13	0.92-4.95	0.0531
IAV	Age	> 3 years	3.20	1.42-733.00	0.0021
	Activity	Breeding and recreation	0.23	0.09-0.55	<0.001
	Place of living	Being stabled	4.00	1.24-13.71	0.0085
	Access to pastures	No having access to pastures	0.20	0.08-0.53	<0.001
*B. caballi*	Access to pastures	Access to pastures	3.76	1.54-9.16	<0.001
*T. equi*	Age	> 3 years	1.92	0.91-4.08	0.0624
*N. caninum*	Age	> 3 years	7.26	0.95-24.8	0.0599
	Activity	Work and sport	3.99	0.79-22.01	0.0489
	Parturition	Having multiple parturition	54.00	1.89-212.32	0.0257

OR: Odds Ratio; CI: 95% Confidence Interval; P: Probability.

## Discussion

None of the horses in the present study tested positive for EIAV, consequently no risk factors could be determined. Presumably, most of the animals that participated in this study were previously tested for EIAV (Sellon and Long, 2007), since they need negative results to be housed in the stables or to participate in competitions.

However, EIAV occurs in Costa Rica and has a wide distribution, as reported by Pineda (1998), who found that 29.5% of participating horses in the Rally of Tilarán were persistently infected and were inapparent carriers of EIAV.

Antibodies to EHV-4 were detected in 96.7% of the animals tested, showing a wide distribution among horse population. No risk factors could be established, due that most of the animals were seropositive, however most (167/175, 95.4%) of the owners of seropositive animals did not notice respiratory disease in their animals which leads us to conclude that the infection may occur subclinically in Costa Rica (Mayr, 1987; Ma *et al.*, 2013). The eight EHV-4 seropositive horses with reported respiratory disease were also seropositive to IAV, which would indicate that it is most likely to be the agent responsible for the clinical presentation and not EHV-4.

On the other hand, only nine (5.0%) animals yielded positive results to EHV-1, a positive correlation between seropositive mares and abortion was determined (p=0.027), which is in agreement with the literature, that reported EHV-1 as the most common equine herpesvirus associated with abortions (Murphy *et al.*, 1999; Sellon and Long, 2007). The EHV-1 seropositive animals were three stallions and six mares, living in the Alajuela (5), San José (3) and Heredia (1) provinces. They were used for recreational (6 mares and 2 males) and breeding purposes (1 male), maintained with other horses, and had access to shared pastures, representing a source of infection for other horses (Murphy *et al.*, 1999).

To our knowledge, this represents the first report of the presence of EHV-1 and EHV-4 in Costa Rica, finding a wide distribution for EHV-4, and limited distribution of EHV-1, which is in accordance with reports from Ataseven *et al*. (2008).

A total of 80 (44.2%) equines showed antibodies to WNV, they were distributed in all provinces (31 in Alajuela, 34 in San José, 12 in Heredia, and 3 in Cartago). Animals >3 years old, mainly used for work and sports, and that had access to pastures had more probability to be seropositive, than younger and stabled horses; results that are in accordance with the epidemiology of the virus (Ulbert, 2011).

The first evidence of WNV activity in Costa Rica was reported by Hobson-Peters *et al*. (2011), who detected in 2004 antibodies in 28% of equines in the Guanacaste province. In 2009, SENASA (National Animal Health Service) reported the first equine case in this province. Our results indicate, that WNV infection occurs silently in equines living in the provinces of the Central Valley of Costa Rica. Possible explanations for reduced disease burden in our horses may be due to the presence of a less virulent WNV strain in Latin America and the Caribbean or due to serological cross-reaction with other related flaviviruses present in the region (Komar and Clark, 2006; Ulbert, 2011).

With respect to Influenza A virus, a total of 41.8% (61/146) equines showed antibodies to IAV, the seropositive animals were distributed in Alajuela (28), San José (24), and Heredia (9). Equines >3 years, used for breeding and recreational purposes, mainly stabled, although with access to pastures were determined with more probability to be seropositive to IAV. These findings are in agreement with the epidemiology of the disease that needs close contact between horses to be transmitted (stabulation), and due, that animals used for breeding and recreational purposes, more often participate in fairs and street exhibitions in Costa Rica, having more close contact to other horses (Murphy *et al.*, 1999).

Although disease occurs throughout the world, there have not been reported outbreaks so far in Costa Rica. However, results of the present study indicate that the virus is distributed in the Central Valley from Costa Rica. Since equines seropositive to IAV also presented antibodies to EHV-4, and eight animals in this group presented respiratory disease, further studies to determine the etiology of respiratory disease in horses from Costa Rica is recommended.

A similar result as EHV-1 was obtained for EVAV, a total of 9 out of 181 (5.0%) equines were seropositive; however no risk factors were determined, due to the small amount of positives. The EVAV seropositive animals were eight males (two of them not castrated) and one mare, most animals (8/9) were older than 3 years, and living in the provinces of Alajuela (3), San José (3), Heredia (1), and Cartago (2). All the horses were used for recreational purposes, and were stabled with other horses. Since most infections of EVAV occur subclinical or asymptomatic, laboratory tests are very important. The detection of two seropositive stallions suggests a risk of dissemination of the virus throughout the country, if control measures are not implemented (Murphy *et al.*, 1999; Sellon and Long, 2007).

A higher seropositivity to *T. equi* (38.1%) than to *B. caballi* (19.9%) was determined, which is in agreement with reports from Posada (2012) and Sellon and Long (2007). This could be due, to the fact that horses infected with *T. equi* remain seropositive and as reservoirs of the parasite for life, whereas horses infected with *B. caballi* are only seropositive for several years (Sellon and Long, 2007). However, horses >3 years and with access to pastures were more likely to be seropositive to *T. equi* and *B. caballi*, respectively, which is in accordance with the fact that equine piroplasmosis is endemic in Costa Rica, due to the presence of tick vectors (Posada, 2012).

A total of 45.8% (83/181) animals were positive to at least one type of piroplasma, and the distribution was as follows: Alajuela (36), San José (27), Heredia (16), and Cartago (4); and 12.1% (22/181) horses showed antibodies against both agents. Outbreaks of clinical disease are uncommon in endemic areas; acute clinical disease is most often observed when naive horses are moved into endemic areas (Posada, 2012).

A total of eight (4.4%) equines showed antibodies to *N. caninum*, six males (four of them castrated) and two mares, all living in the San José province. Animals >3 years old, used for work or sports had more probabilities to be seropositive to *N. caninum*, also mares with at least one delivery. Further studies are required to determine whether these animals were infected with *N. caninum* or *N. hughesi* (Dangoudoubiyam *et al.*, 2011). Using molecular techniques, *N. caninum* was recently detected in cases of abortion and neonate mortality (Leon *et al.*, 2012); also, seroprevalences of *N. caninum* had been reported previously in the United States of America (Sellon and Long, 2007).

Finally, seven out of 146 (4.8%) horses were found to be seropositive to *C. abortus*, a relatively low seroprevalence. No risk factors were established due to the small size of the positive animals. The *C. abortus* seropositive animals were six males, two of them castrated, and one mare. All horses were >3 years old and living in the Alajuela (2), San José (3), and Heredia (2) provinces. Two males were used for breeding, three for recreational, and one for work purposes. The mare was used for sports. The animals were held stabled with other horses, and only three had access to shared pastures. This is the first report of *C. abortus* seropositivity in horses from Costa Rica, involvement of this agent in abortions need further investigation in future studies.

Almost all studied agents in the present study are notifiable to the International Organization of Epizooties, with exception of EHV-4 and *N. caninum*. Actually, only diseases caused by EIAV, WNV and Piroplasmosis were reported to be present in Costa Rica.

However, presence of possible carriers of EHV-1, EHV-4, EVAV, and *C. abortus* in horses of the Central Valley of Costa Rica were determined, also, that WNV seems to be disseminated among the horse population silently. Veterinarians are recommended to take into account these infectious agents in the diagnosis and differential diagnosis of respiratory and reproductive diseases in horses.

### Conflict of interest

The Authors declare that there is no conflict of interest.
